# Interfacial Thermal Resistive Switching in (Pt,Cr)/SrTiO_3_ Devices

**DOI:** 10.1021/acsami.3c19285

**Published:** 2024-03-13

**Authors:** Víctor Álvarez-Martínez, Rafael Ramos, Víctor Leborán, Alexandros Sarantopoulos, Regina Dittmann, Francisco Rivadulla

**Affiliations:** † Centro Singular de Investigación en Química Biolóxica e Materiais Moleculares (CIQUS), Universidade de Santiago de Compostela, 15782 Santiago de Compostela, Spain; ‡ Departamento de Química-Física, Universidade de Santiago de Compostela, 15782 Santiago de Compostela, Spain; § Peter Gruenberg Institute (PGI-7) Forschungszentrum Juelich GmbH and JARA-FIT, 52425 Juelich, Germany

**Keywords:** thermal resistive switching, ionic devices, thermal conductivity, active interfaces, memristor, thermal memories

## Abstract

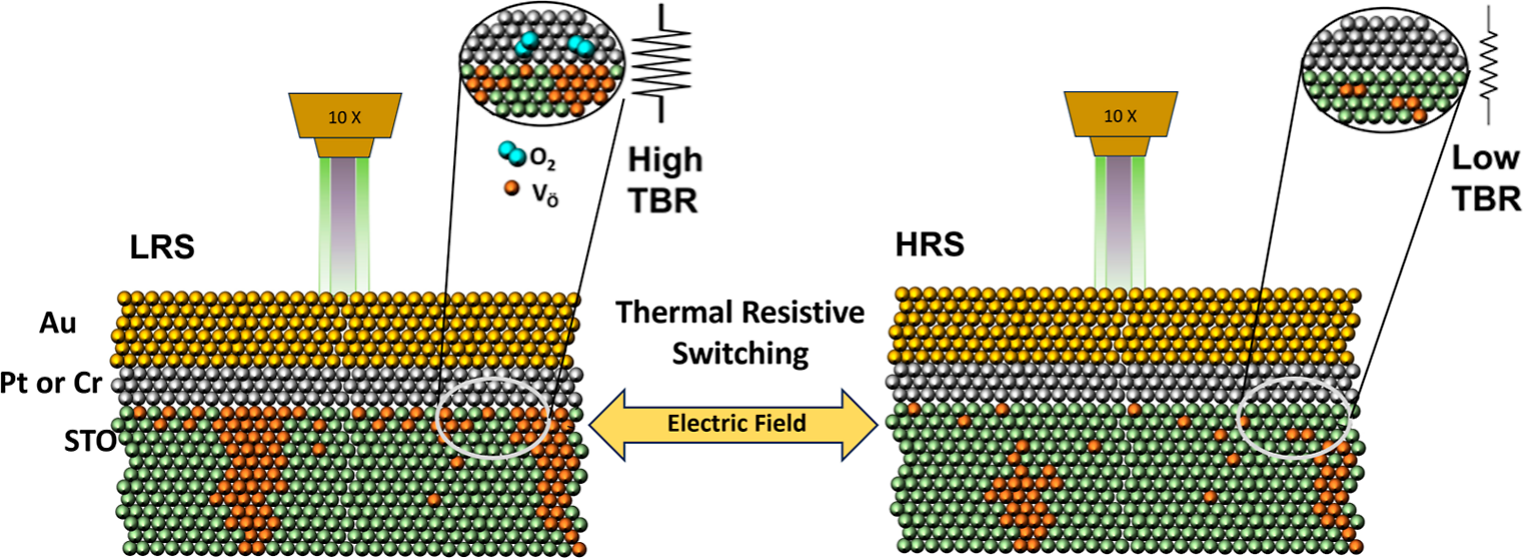

The operation of oxide-based memristive devices relies on the fast accumulation and depletion of oxygen vacancies by an electric field close to the metal–oxide interface. Here, we show that the reversible change of the local concentration of oxygen vacancies at this interface also produces a change in the thermal boundary resistance (TBR), i.e., a thermal resistive switching effect. We used frequency domain thermoreflectance to monitor the interfacial metal–oxide TBR in (Pt,Cr)/SrTiO_3_ devices, showing a change of ≈20% under usual SET/RESET operation voltages, depending on the structure of the device. Time-dependent thermal relaxation experiments suggest ionic rearrangement along the whole area of the metal/oxide interface, apart from the ionic filament responsible for the electrical conductivity switching. The experiments presented in this work provide valuable knowledge about oxide ion dynamics in redox-based memristive devices.

## Introduction

Strategies for controlling the heat flow in solids comprise the use of artificial interfaces,^1−5^ domain walls in ferromagnets and ferroelectrics,^6−9^ anisotropic mass distribution in nanowires,^10^ or point defects in crystalline solids,^11−13^ among others.^14^ On the other hand, achieving a dynamic manipulation of heat transport implies the design of reconfigurable thermal states,^15−19^ which poses a much bigger challenge, but it is essential for dealing with thermal energy management in electronics and other energy-demanding technologies.^20^ Ionic electrochemical intercalation^21−23^ has stood out as a possibility in recent years, although the slowness of switching and the use of an ionic liquid/gel phase limit its applications.

However, the relatively large O^2–^ ion mobility in several transition-metal oxides enables the change of the local oxygen concentration with an electric field, at a fast switching speed, in simple two-terminal metal/oxide/metal devices.^24^ This is the principle of the resistive switching (RS) effect: the reversible switching between a high and a low electrical resistance state (HRS and LRS, respectively) of a dielectric, with an electric field.^25^ In oxides, RS usually occurs through the formation of conducting filaments of oxygen vacancies between the metal electrodes, and its accumulation close to the metal–oxide anodic interface.^26,27^

Given the strong effect of oxygen vacancies on the thermal conductivity of oxides,^12,13,28,29^ we hypothesize that, associated with the electrical RS effect, there should be a change in the thermal resistance of the metal/oxide interface: a thermal RS effect. Studying the effect of oxygen vacancies on the thermal resistance can contribute to the understanding of the heat transport phenomena across interfaces in all-solid-state devices with potential applications for energy harvesting.^30^

On the other hand, the sensitivity of the RS effect to the oxygen partial pressure and humidity^27,31,32^ has revealed the important role of metallic electrodes as storage and ion/molecular conductors, beyond being mere surfaces for electronic transfer. The exchange of ions across the metal/oxide interface and the microstructural stability of the interface itself in the LRS and HRS implies an ionic redistribution,^33^ which could also affect the interfacial thermal resistance.

Therefore, the goal of this work is 2-fold: (i) to probe and quantify the existence of a thermal RS effect, associated with the electrical RS, and (ii) to study the oxide-ion dynamics close to the interface for a better understanding of the RS phenomenon itself.

## Experimental Details

Two types of devices were studied in this work.

(i) (Pt, Cr)/STO devices: epitaxial thin films of SrTiO_3_ (STO) ≈35 nm thick, were grown by pulsed laser deposition on (001)-Nb(0.5 wt %):SrTiO_3_ substrates. The films were deposited at 765 °C, P(O_2_) = 100 mTorr, and cooled down to room temperature at 5 °C/min under the same atmosphere. X-ray diffraction analysis shows a lattice parameter consistent with stoichiometric deposition (see Figure S1 in the Supporting Information). Top metallic electrodes of (60/5) nm thick Au/Pt and Au/Cr with sizes ranging from 10 × 10 to 600 × 600 μm^2^ were deposited for *I*–*V* curve testing. Pt or Cr is in direct contact to STO and forms the metal/oxide interface. Au is deposited on the top of Pt or Cr to serve as a thermal transducer for optical thermal conductivity measurements, as explained below.
(ii) (Pt, Cr)/Nb:STO devices: in this case, the metallic Au/(Pt,Cr) electrodes are deposited directly on the top of a (001)-Nb(0.5 wt %):SrTiO_3_ substrate, which has been previously annealed at *T* = 765 °C and P(O_2_) = 100 mTorr for 2 h. Thermal annealing induces the segregation of SrO at the surface, resulting in a robust RS effect (Figures S4 and S5, Supporting Information).^34^ As before, the active interface is made of either Cr or Pt in direct contact to Nb:STO.

The electrical characterization was performed at room temperature in air under atmospheric conditions. The devices were cycled between the LRS and HRS (*V*_SET_ = 2.5 V/*V*_RESET_ = −5 V) with a step of 10 mV and a 30 mA current compliance (CC). The forming process was performed under the same conditions of the step and CC, but to a higher voltage, *V*_form_ = 3 V.

Thermal conductivity and thermal boundary resistance (TBR) were measured by frequency domain thermoreflectance (FDTR) using the 60 nm thick layer of Au as a transducer.^35−37^ In this optical technique, the phase lag between a pump and a probe laser is fitted to an analytical solution of the heat diffusion equation to obtain the thermal properties of the sample (see the Supporting Information for further details of the setup and fittings; Figures S6–S9).

## Results and Discussion

**Figure 1:**
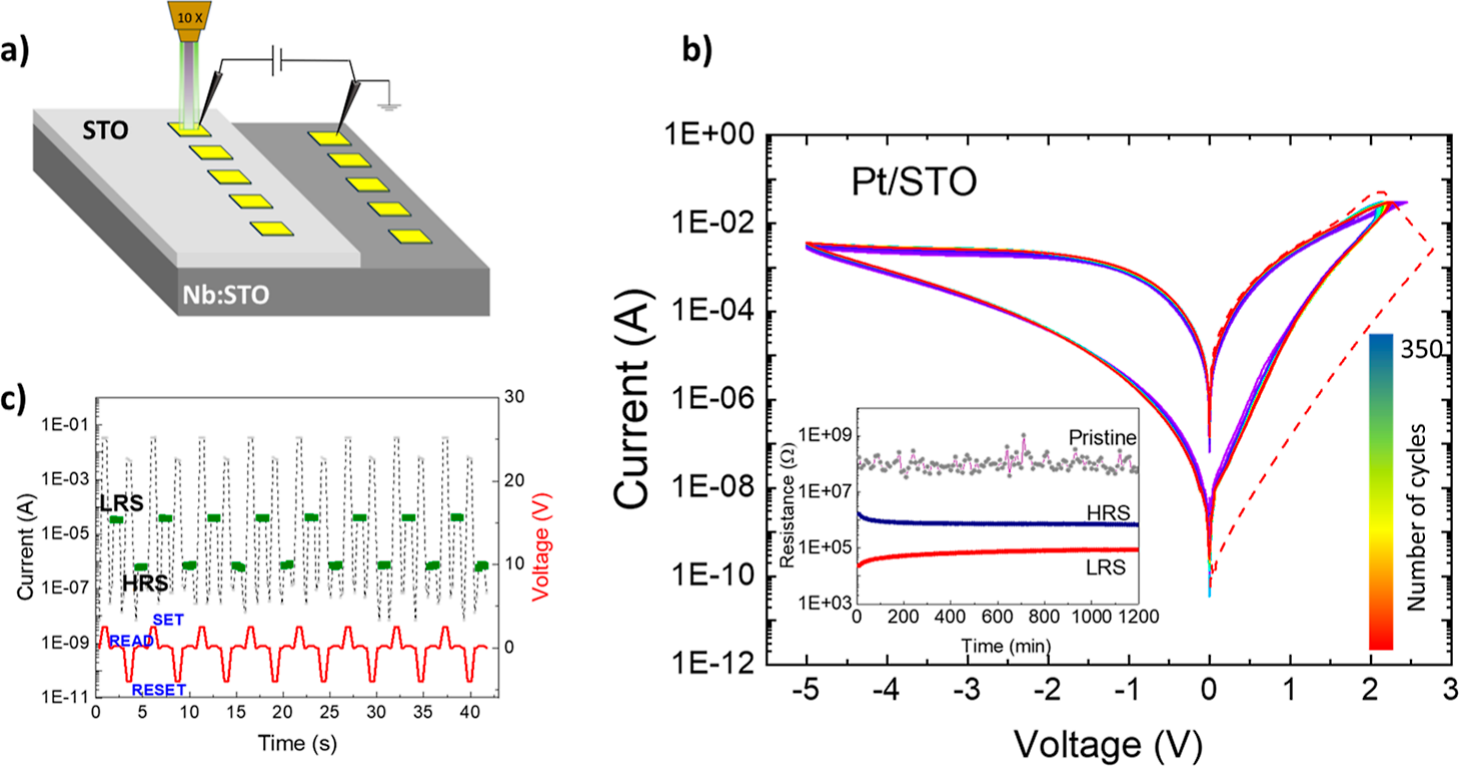
(a) Schematic representation of the (Pt, Cr)/STO devices used in this work: epitaxial thin films of SrTiO_3_ ≈ 35 nm thick were deposited on the top of a (001) Nb(0.5 wt %):SrTiO_3_ single crystal, covering half the surface. (60/5) nm thick Au/Pt and Au/Cr contact pads of sizes ranging from 10 × 10 μm^2^ to 600 × 600 μm^2^ were deposited for *I*–*V* curve testing. Interfacial thermal resistance was measured after the SET/RESET operation by FDTR, focusing the pump/probe lasers with an objective on the surface of the pads. The spot radius of the pump laser was changed in the range 1/*e*^2^ ≈ 4–11 μm. (b) *I*–*V* curves of one of the devices tested in this work: applying a positive voltage to the Pt electrode (SET) drives the system to the LRS, while a negative bias (RESET) switches it to a HRS, in a fully reversible process. For the reproducibility and endurance test, 350 SET/RESET consecutive sweeps were performed, and voltages for SET and RESET were *V*_SET_ = 2.5 V and *V*_RESET_ = −5 V, respectively, with a step of 10 mV and a 30 mA CC. The red dashed line shows the forming process of the conducting filaments, and the inset shows the stability experiment for the pristine state, HRS, and LRS for a Au/Pt/STO device. Electrical resistance was read every 10 min for 20 h, with *V*_READ_ = 100 mV. (c) Voltage pulses performing SET/RESET and reading of the corresponding resistance states (LRS and HRS) demonstrate the excellent electrical performance of our devices.

Figure 1 shows the RS performance of a representative Pt/STO device. After an initial forming process, the device can be switched between an LRS (SET voltage +2.5 V) and an HRS (RESET voltage −5 V) hundreds of times without appreciable degradation (Figure 1b,c); similar results were obtained for Cr/STO (Supporting Information, Figure S2). In the measurements, the sign corresponds to the voltage at the top metallic electrode, and the bottom electrode is electrically grounded (Figure 1a). The device is stable under ambient conditions for days at 0 V, with an ON/OFF resistance ratio ≈10.

We did not observe any dependence of the resistive states on the size of the top metallic electrodes, at least in the range studied in this work, from 10 × 10 to 600 × 600 μm^2^ (Supporting Information, Figure S3).

**Figure 2:**
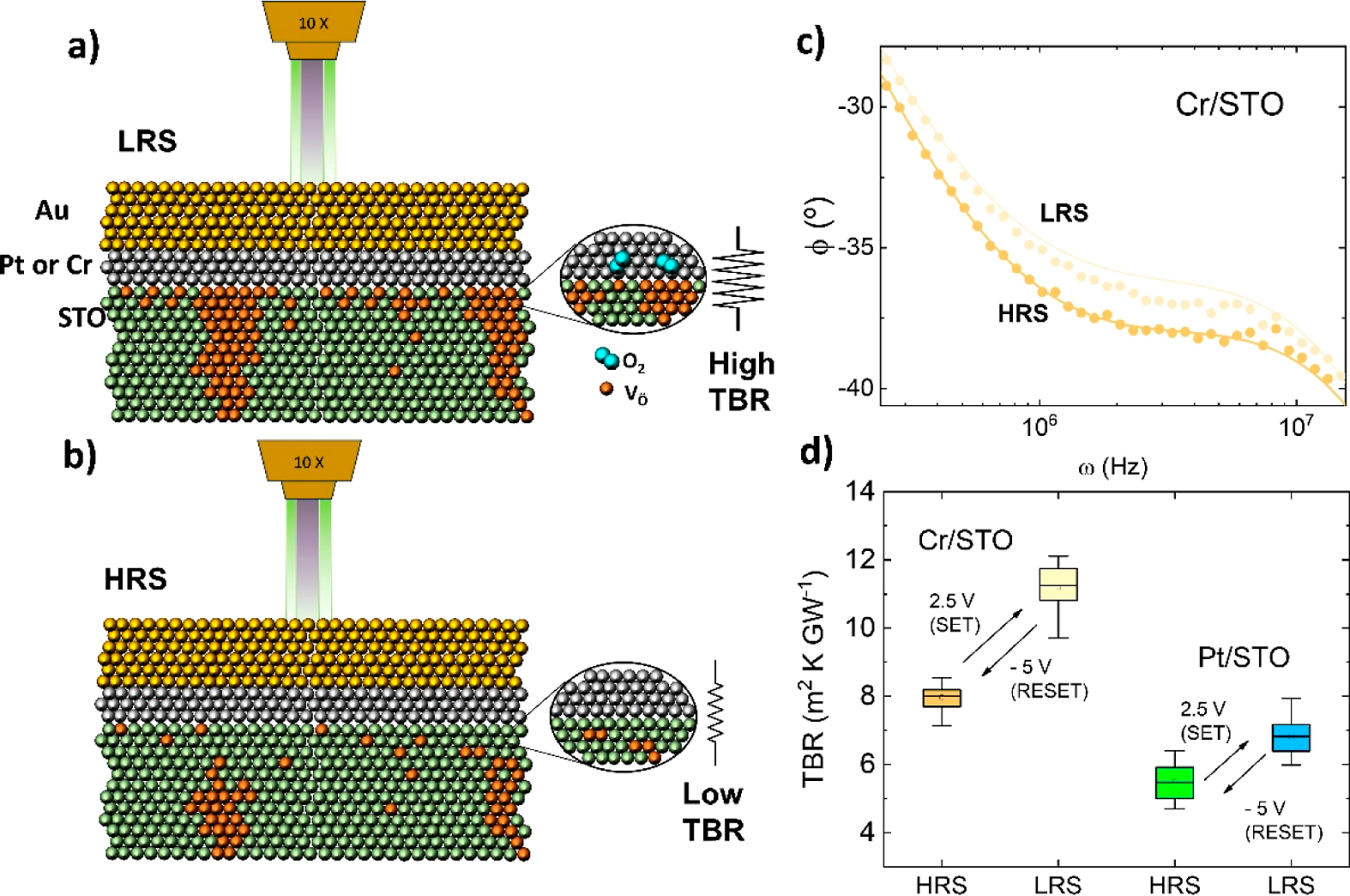
Proposed microstructure of the (Cr,Pt)/STO interface in the LRS (a) and HRS (b), following Cooper et al.^27^ In the LRS, oxygen vacancies and interstitial molecular O_2_ accumulate in the oxide thin film and in the Pt electrode close to the interface, respectively. In the HRS, O_2_ moves back from the metal electrode into the film, filling the vacancies and restoring the structure of the interface. (c) Example of phase vs frequency curves in FDTR experiments. Filled circles are the experimental data for the HRS and LRS, and the lines show the fitting to the thermal transport model (see the text). (d) Reversible change in the interfacial TBR for Cr/STO and Pt/STO interfaces, after cycling the device between the HRS and LRS. Despite the different absolute values of the TBR of the Cr/STO and Pt/STO interfaces, the ON/OFF ratio is ≈ 20% in both cases.

As explained before, the thermal properties of the devices were obtained by fitting the phase data of FDTR experiments on each metallic pad to an analytical solution of the heat diffusion equation in a multilayer model.^36^ An example of experimental ϕ(ω) data, representative of the HRS and LRS, is shown in Figure 2c. For minimizing the number of fitting parameters in the model, we determined most of them from independent experiments so that the TBR of the metal/oxide interface is the only free parameter of the fitting (see the Supporting Information for further details).

For a reliable statistical determination of the TBR, 40 different ϕ(ω) curves were acquired on random points of each Au/(Pt,Cr) pad. The measurements were done on 55 × 55, 71 × 71, and 600 × 600 μm^2^ pads, without appreciable differences. Several pads were tested in each resistive state so that between 100 and 200 phase shift curves were measured and fitted to the thermal model to estimate the statistical distribution of TBR in the LRS and HRS of a single device. A low heating power of 1 mW was used in the experiments to avoid temperature-induced migration of the interfacial vacancies during the experiment. The main results of the analysis of the FDTR experiments are shown in Figure 2d. Some important observations can be made from these data.

(i) It is possible to induce a reversible change of the metal/oxide TBR, with an electric field, associated with the electrical HRS and LRS.
(ii) Although the absolute value of the TBR is larger for the Cr/STO interface than for the Pt/STO interface, the relative change upon electric field switching is ≈ 20–25% in both cases. The actual values of TBR can be influenced by the assumption of a constant κ(STO-film) ≈ 2 Wm^–1^ K^–1^ (see Table S1 in the Supporting Information) independent of the electrical state of the sample (HRS or LRS).^38^ However, as shown in Figure 2c, there is a large change in the bare ϕ(ω) curves of the HRS/LRS, which supports a variation of TBR between both states.
(iii) The high TBR (H-TBR) and low TBR (L-TBR) are related to the electrical LRS and HRS, respectively. The larger TBR in the LRS implies a more defective interface than in the HRS.

Regarding point (iii), Cooper et al.^27^ detected a substantial increase of Ti^3+^, associated with the accumulation of oxygen vacancies, V_Ö_, close to the Pt/STO interface under a positive bias (LRS). Moreover, the sensitivity of the resistive state to the oxygen partial pressure in the surrounding atmosphere led several authors to conclude that oxygen redistribution within the active oxide layer cannot be the only source of RS, but oxygen has to move across the metal–oxide layer during device operation.^27,31,39^ Thus, under a positive bias, anodic oxidation of lattice oxide ions in STO occurs, which are removed as molecular oxygen according to^27^
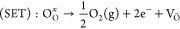
1

This results in a large accumulation of V_Ö_ close to the metal–oxide interface and of molecular oxygen at the Pt grain boundaries (Figure 2a).^39,40^ Both mechanisms imply the concentration of defects close to the metal/oxide interface, which increases the TBR in the LRS.

On the other hand, O_2_ reincorporates into the oxide lattice at a negatively biased Pt/STO interface
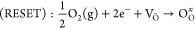
2

This restores an interface in the HRS more like that of the pristine state (similar to its state before switching) and lowers the interfacial thermal resistance (Figure 2b).

Therefore, the switching of the interfacial TBR reported here supports the scenario of O_2_/V_Ö_ exchange across the metal/oxide interface in (eightwise) bipolar RS devices.^41^ The reversibility of the TBR indicates that the evolution of oxygen and reincorporation does not result in delamination of the Pt electrode or irreversible damage of the Pt/STO interface, at least in the conditions explored in this paper.

**Figure 3:**
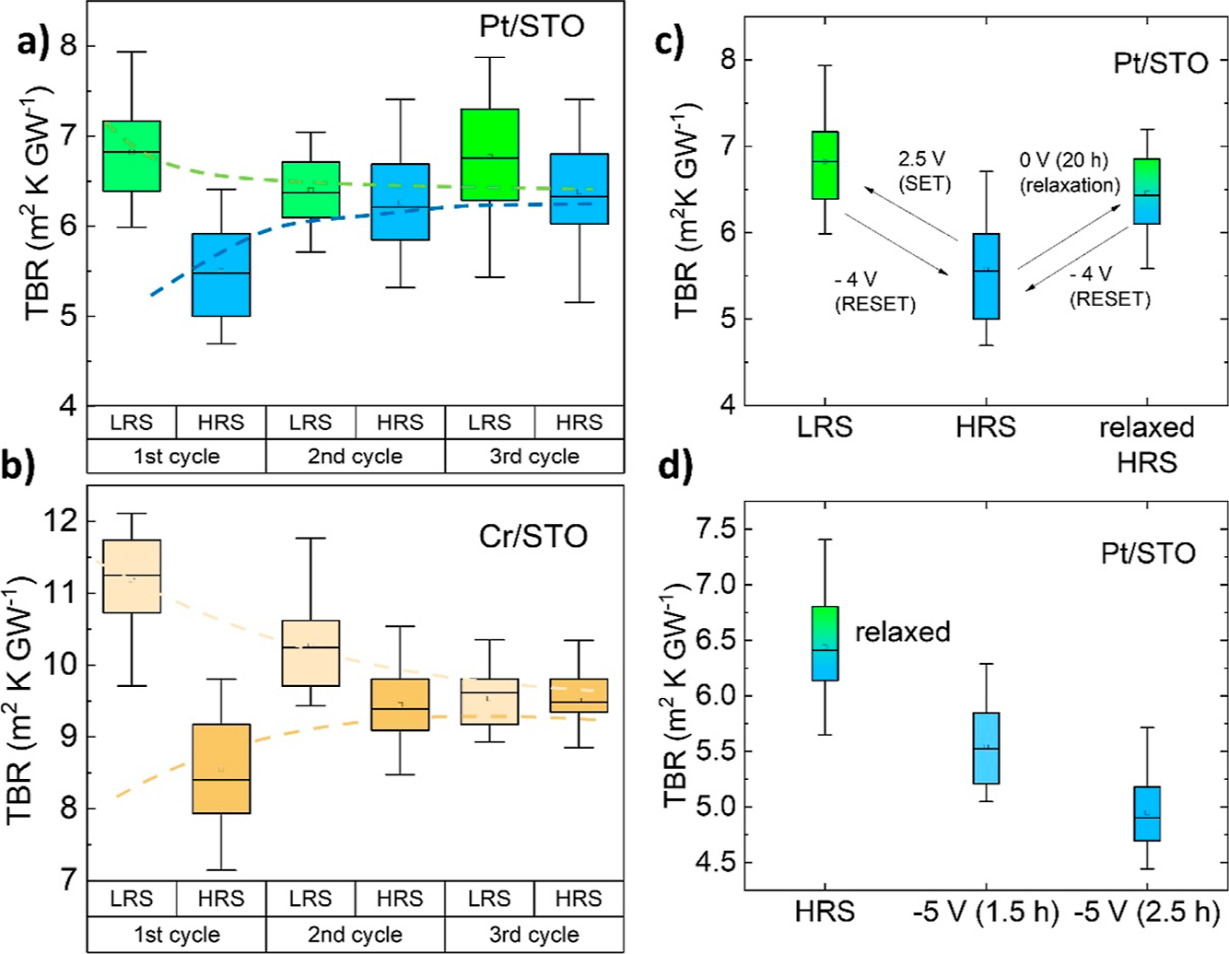
TBR of the Pt/STO (a) and Cr/STO (b) interfaces during consecutive SET and RESET cycles, which drives the device to the LRS and HRS, respectively. After 3 consecutive cycles, the TBRs of the HRS and LRS are statistically equal, with a 99.99% significance level. (c) After 20 h at 0 V, the TBR increases up to a value like the LRS and recovers the tunability of the thermal device, which can be cycled again. (d) Forced diffusion of oxygen vacancies (increasing voltage or time) produces a larger effect over the TBR of the Pt/STO interface; similar effects were found in Cr/STO interfaces.

In Figure 3 we show the evolution of the TBR after switching the device several consecutive times between the HRS and LRS. The difference between H-TBR and L-TBR reduces after 3–4 voltage cycles, where it reaches an intermediate value (Figure 3a,b). However, letting the device relax at 0 V in the HRS results in a gradual increase of the TBR, and after 20 h, it reaches the value of H-TBR, despite remaining in the state of high electrical resistance (Figure 3c). Applying a RESET voltage reduces the TBR (Figure 3c,d), and the device can be switched between the L-TBR and the H-TBR again.

These experiments suggest that a RESET voltage induces a depletion of V_Ö_ close to the Pt/STO interface; relaxation of V_Ö_ at 0 V against a chemical potential gradient recovers some vacancies close to the metal/oxide interface. This process of relaxation occurs in the HRS, and it is consistent with the change in its electrical resistance from 1.72 MΩ, immediately after RESET, to 0.72 MΩ, after 20 h (inset to Figure 1). However, the device remains in the HRS, with a large ON/OFF ratio. Therefore, while the main cause of electrical RS is the formation/destruction of stable conducting filaments of V_Ö_ across the oxide, the switching of the thermal resistance is caused by the accumulation/depletion of V_Ö_ at the metal/oxide interface.

The possible effect of the redistribution of oxygen within the metal electrode will have a small contribution to the reported TBR due to the much lower sensitivity of our experiment to small changes in the thermal conductivity of the metallic electrode.

These relaxation experiments are consistent with the homogeneous accumulation/depletion of V_Ö_ along the Pt/STO interface (responsible of TBR switching), besides the formation/breaking of a conducting filament across the oxide (responsible of electrical RS).^42^

Similar TBR switching and relaxation was observed in Cr/STO devices, which suggests a similar mechanism of oxygen exchange across the metal/oxide interface. The ability of Cr_2_O_3_ to support a large concentration of diffusive oxygen vacancies^43^ and the observation of RS in metal/Cr_2_O_3_ devices^44^ point toward the existence of a layer of this oxide close to the STO film. Moreover, it has been reported that Cr_2_O_3_ has a work function similar to that of Pt,^45,46^ inducing the formation of a Schottky barrier at the metal/STO interface and the presence of a RS effect.

**Figure 4:**
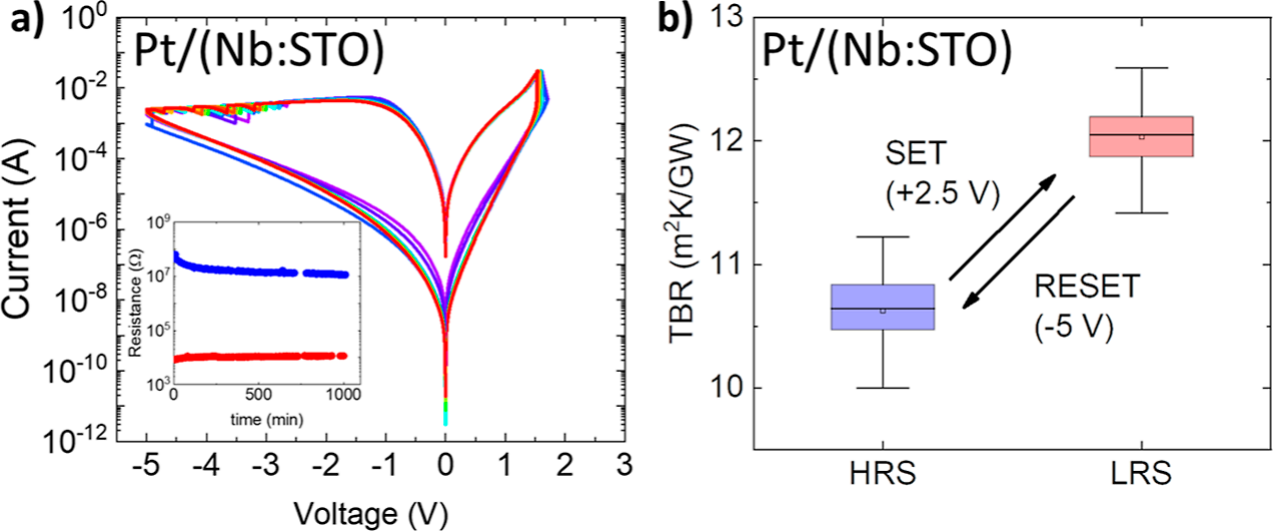
(a) *I*–*V* curves for a Pt/Nb:STO device tested in this work. The Nb:STO single crystal was previously annealed at 765 °C (2 h) under P(O_2_) = 100 mTorr before the deposition of Pt. A positive (negative) SET (RESET) voltage applied to the Pt electrode drives the system to the LRS (HRS) in a fully reversible process. The inset shows the stability experiment for the device; the electrical resistance was read every 10 min for 15 h, with *V*_READ_ = 100 mV. (b) TBR switching of the Pt/Nb:STO interfaces after SET/RESET voltage. As for the other devices, at least 40 different ϕ(ω) measurements were performed on the surface of each pad and fitted for a statistical distribution of the TBR in each electrical state. The ON/OFF ratio is ≈ 10% in this case.

Finally, to discard any possible influence of the microstructure of the film on the existence of the interfacial thermal RS effect, we tested the thermal RS of (Cr,Pt)/Nb:STO devices. In this case, the metal electrode is deposited directly on the top of a Nb:STO single crystal that has been annealed at 765 °C (2 h) under P(O_2_) = 100 mTorr. The interface between Pt and annealed Nb:STO presents a robust RS effect (see Figures 4a and S4 in the Supporting Information).

The TBR measured in the two resistive states is shown in Figure 4b for a Pt/Nb:STO device. As in the Pt/STO/Nb:STO devices, there is a reversible switching between the L-TBR and H-TBR, associated with the electrical HRS and LRS, respectively. The effect is smaller, however, on the order of 10–12%.

## Conclusions

In this paper, we have experimentally demonstrated the existence of a thermal RS effect in RS devices. The results support the hypothesis of O_2_/V_Ö_ exchange across the metal/oxide interface in (eightwise) bipolar RS devices. Although with different intensities, the switching of the interfacial TBR was observed in two types of devices (with and without the STO film) and with two different interfaces, (Pt,Cr)/STO, pointing to a general behavior. The magnitude of the change in the TBR is too small for thinking about any practical application at this stage, at least with STO devices, and requires further optimization. However, Joule heating is crucial in assisting the RS effect in resistive random-access memories; therefore, having the actual values of TBR will be very important for optimizing the performance of these devices. The extreme sensitivity of thermal conductivity to point defects makes it a very valuable technique for the investigation of the V_Ö_ relaxation occurring close to the metal–oxide interfaces, providing important information for the understanding of ion dynamics in RS devices.

## Supporting Information Available

Structural characterization of the films (X-ray diffraction and reflectivity analysis); details of the FDTR measurements and analysis, as well as the independent determination of the parameters entering the thermal model; and comparison of the different interfaces in the RS effect and its areal dependence (PDF)
