# Supplementary material for: Interfacial Thermal Resistive Switching in (Pt,Cr)/SrTiO3 Devices
Source: ACS Appl Mater Interfaces. 2024 Mar 13;16(12):15043–9. doi: 10.1021/acsami.3c19285 (PMC10982933; doi:10.1021/acsami.3c19285)
Supplement: Supplementary file 1 — am3c19285_si_001.pdf [file am3c19285_si_001.pdf]

Supporting Information for:

Interfacial Thermal Resistive Switching in (Pt,Cr)/$SrTiO_3$ devices.

Victor Álvarez-Martínez,[1,2] Rafael Ramos,[1,2*] Victor Leborán,[1] Alexandros Sarantopoulos,[3] Regina Dittmann,[3] Francisco Rivadulla.[1,2*]

[1]Centro Singular de Investigación en Química Biolóxica e Materiais Moleculares (CIQUS), Universidade de Santiago de Compostela, 15782-Santiago de Compostela, Spain.

[2]Departamento de Química-Física, Universidade de Santiago de Compostela, 15782-Santiago de Compostela, Spain.

[3]Peter Gruenberg Institute (PGI-7) Forschungszentrum Juelich GmbH and JARA-FIT 52425 Juelich, Germany.

Structural characterization

The structural quality of the films was determined by X-ray diffraction (XRD), and the thickness by X-ray reflectometry (XRR). Fig. S1a shows the result of a ω-2Θ scan around the Nb:STO(002) peak, illustrating the almost perfect lattice matching condition between film and substrate. Fig. S1b shows the XRR scan for a STO film co-deposited on a SiO_2/Si substrate used for the determination of the STO thin film thickness. Figs. S1c demonstrate the negligible structural effect of post-annealing at $p(O_2)$ = 10 mTorr on a film deposited at $p(O_2)$ = 100 mTorr of O_2.

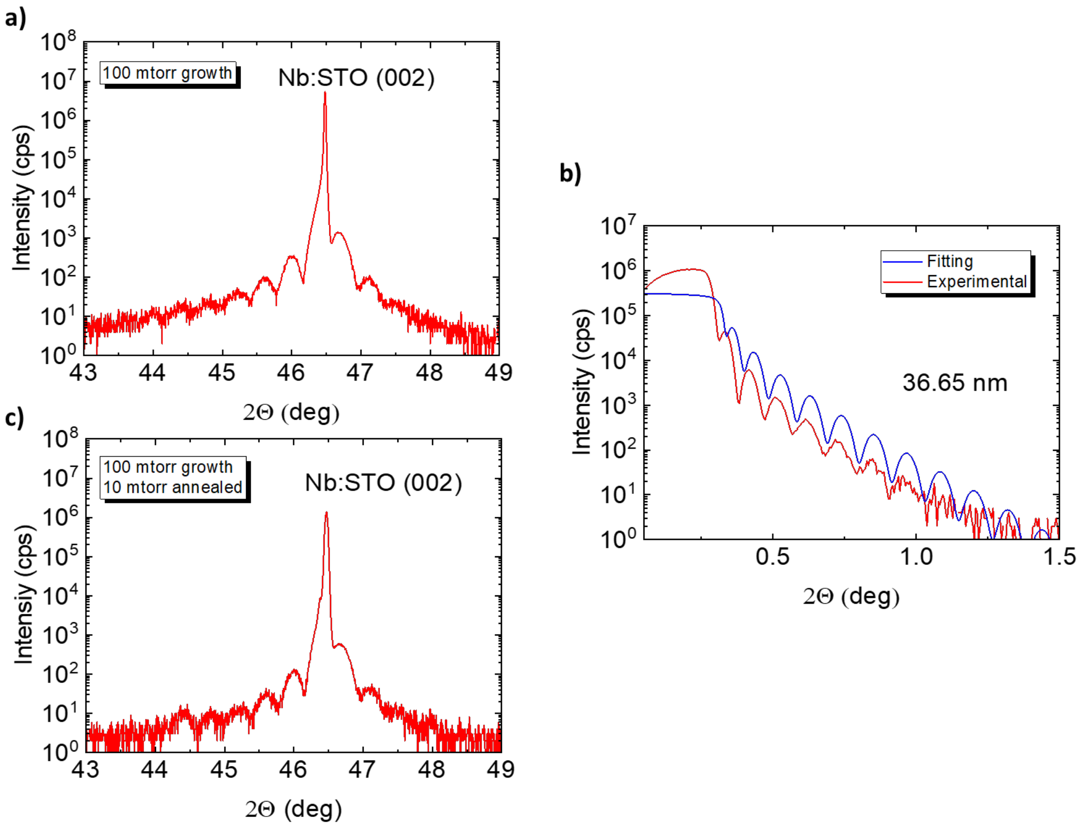

Figure S1. a) XRD pattern of a STO thin film deposited on (001)-Nb:STO single crystal at 100 mtorr oxygen pressure and 765ºC. b) XRR curve for a film co-deposited on a Si substrate, along with the fitting to obtain the

film thickness. c) XRD of the same film in a) after annealing at 765ºC for 2h, and $P(O_2)$=10 mtorr. No structural changes were observed in the XRD diffractogram after the annealing.

Effect of the metal/STO interface on the Resistive Switching behavior

Figure S2 shows typical I-V curves for Cr/STO/Nb:STO and Au/STO/Nb:STO devices. The Cr/STO/Nb:STO devices (Fig. S2a)) show persistent resistive switching and high reproducibility of the I-V curves, after several repeated cycles. On the other hand, the resistive switching (RS) properties of the Au/STO/Nb:STO devices (Fig. S2b)) are quickly degraded after a few cycles.

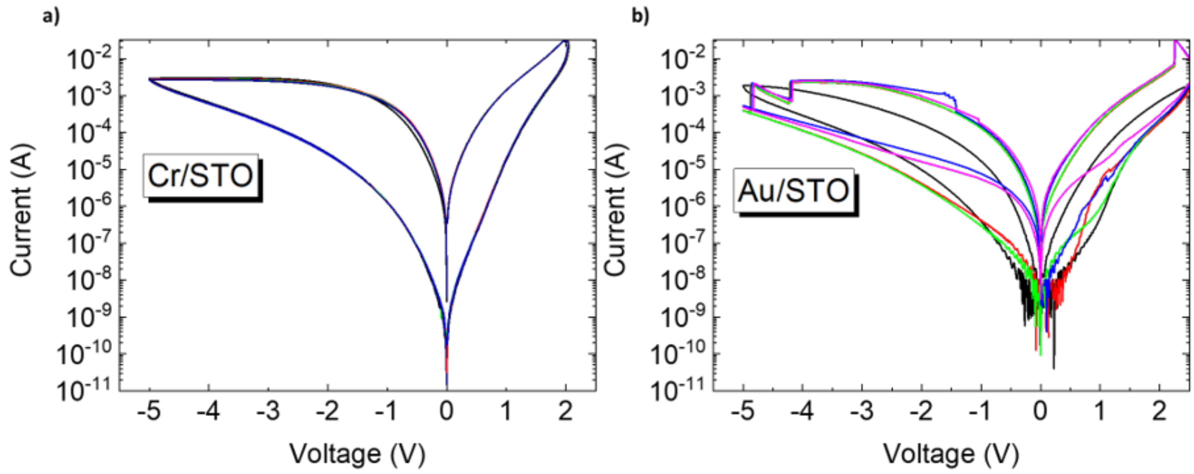

Figure S2. I-V curves were performed on the same metal/STO/Nb:STO device, for **a)** metal=Cr and **b)** metal=Au. For Cr/STO interfaces, the behavior is very similar to Pt/STO devices described in the main text of the paper. For Au/STO interfaces, we observed a rapid degradation of the RS effect, after a few cycles.

Area dependence of the resistive switching in STO films

We studied the dependence of the RS on the area of the metal/oxide interface. Metallic pads with lateral dimensions ranging from 10x10 to 600x600 μm^2 were fabricated by optical lithography and lift-off. As shown in Fig. S3a and S3c the resistance of the HRS and LRS in both (Au/Pt)/STO/NbSTO and (Au/Pt)/NbSTO devices do not show a clear dependence on the area of the pads, at least for the dimensions studied in this work. These results suggest that the electrical switching is due to the formation of filaments of oxygen vacancies joining the top/bottom metallic electrodes. In Fig. S3b we show an actual picture of the top electrodes patterned on a sample, as employed for the experiments described in this work.

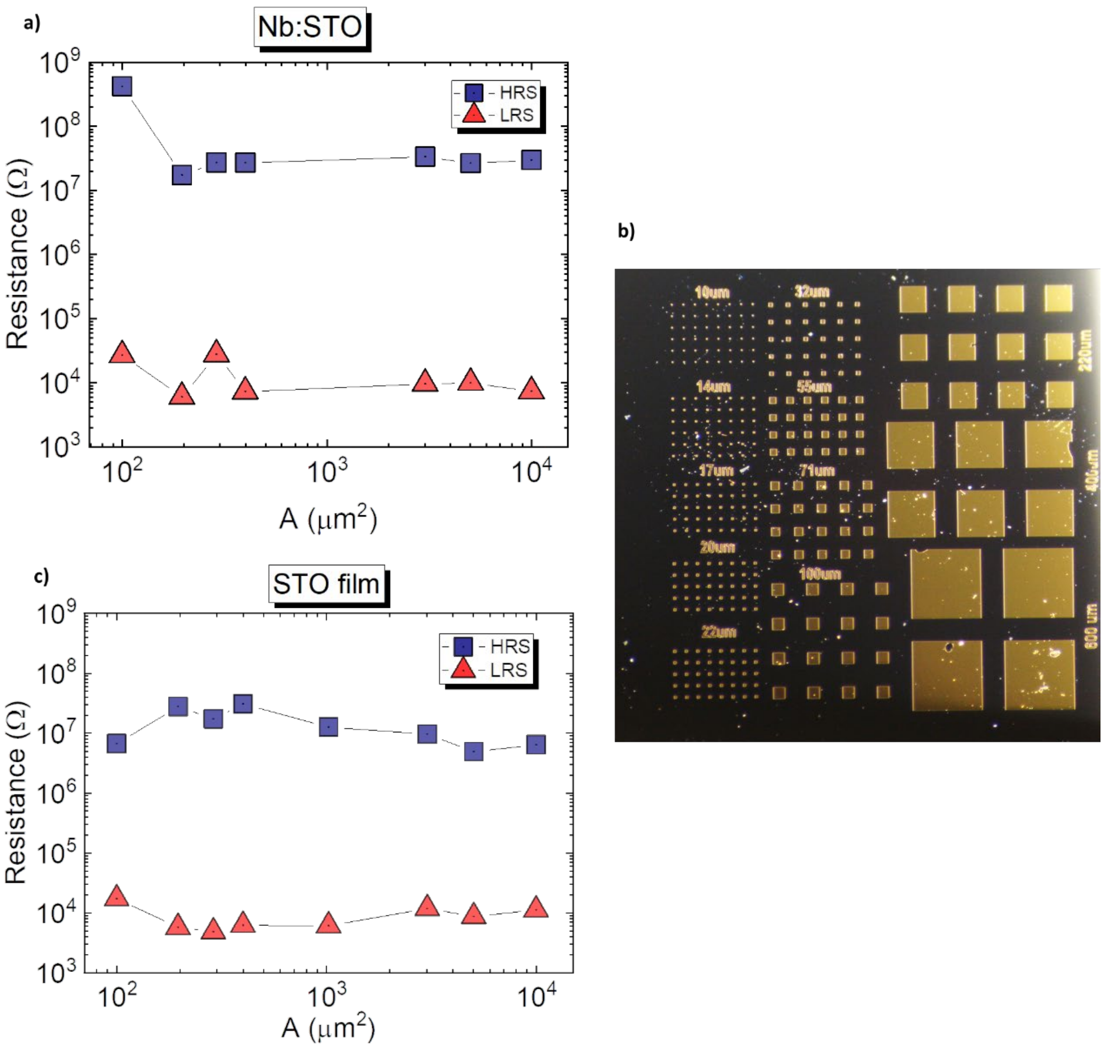

Figure S3. Size dependence of the two different states (HRS and LRS) with the area of the metal pads in a) (Au/Pt)/Nb:STO and c) (Au/Pt)/STO/Nb:STO devices. b) Photography taken from a sample with the pattern of the area dependence experiment. The size of the top electrodes range from 10x10 to 600x600 μm^2.

Effect of annealing on Nb:STO (0.5 wt%) substrates

Figure S4 shows the electrical characterization of metal/Nb:STO devices. Here, (Pt,Cr)/Au (5/60) nm electrodes were directly deposited on the Nb:STO substrates which were previously annealed under different conditions. Figs. S4a show the small irreversibility of the I-V curves using a pristine Nb:STO substrate, and Fig S4b the absence of HRS/LRS retention. Figs. S4c, S4d show the same results for a Nb:STO substrate previously annealed under $P(O_2)$ = 100 mTorr at 765 ºC for 2 h., showing the RS and ON/OFF ratio ≈ 10^3-10^4, with good retention.

We also evaluated the topography of the substrates by atomic force microscopy measurements. Figure S4 shows the results for pristine (Fig. S4e) and annealed (Fig. S4f) Nb:STO substrates, it can be seen that the annealing promotes the formation of SrO particles as reported elsewhere, which are known to improve the resistive switching performance of $SrTiO_3$ devices [Ref. 34 in manuscript]

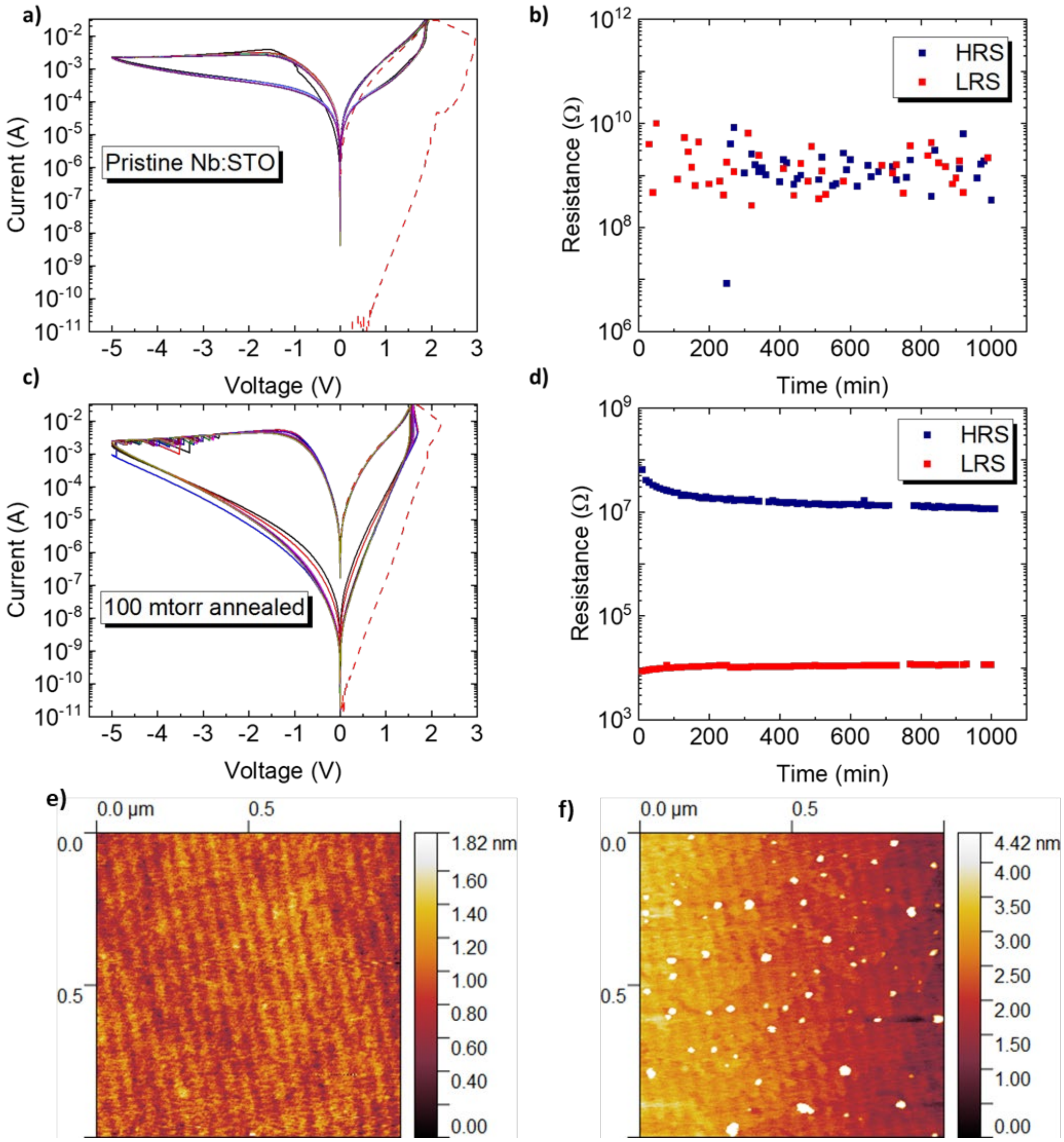

Figure S4. a) I-V curves were performed for pristine Nb:STO (0.5 wt%)/Pt interfacial device. Small reversibility and no retention (b) of HRS/LRS were observed. c) After performing a 765ºC, 100 mtorr oxygen anneal there is both forming (red dashed line) and hysteresis behavior observed in I-V curves as well as in the pristine device. d) Also, there is a high ON/OFF ratio (3-4 magnitude orders) and a resistive state retention over time. The bottom panels show the AFM topography (1×1 μm) of a e) pristine Nb(0.5 wt%):STO and the same substrate after annealing f) at 765ºC and 100 mTorr oxygen pressure for 2 hours. The latter were used in the resistive switching experiments.

Sheet resistivity and Hall measurements.

To test the effect of the thermal annealing in the electric properties of Nb(0.5 wt%):STO substrates, we measured the temperature dependence of the electrical sheet resistivity and the room temperature Hall effect for a pristine Nb:STO and annealed Nb:STO substrate (Fig. S5).
The experiments were performed using the van der Pauw technique in 5x5 mm^2 substrates, with a thickness of 0.5 mm. The carrier density $n = (1.5 \pm 0.1) \times 10^{20}$ cm^{-3} was obtained from the Hall coefficient of the ρ_{xy} vs H curves, with negligible differences between the samples. The value of n is in very good agreement to the expected carrier concentration for 0.5 wt% Nb-doped STO ($n = 1.6 \times 10^{20}$ cm^{-3}).
Therefore, we can infer the annealing affects the surface of Nb:STO, but the bulk transport properties remain unaffected.

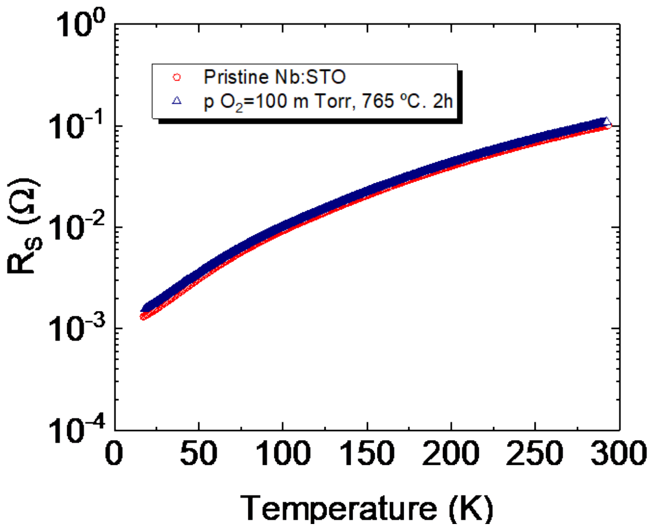

Figure S5. Temperature dependence of the sheet resistance, R_s, for a 0.5 mm-thick Nb:STO substrate, before (open circles) and after (triangles) thermal annealing at 765ºC, 100 mTorr, 2h.

Independent determination of the thermal conductivity and heat capacity of STO substrate.

The thermal conductivity of STO and Nb:STO single crystal substrates was measured by steady-state thermal conductivity measurements. For these experiments, a 100 Ω ceramic resistor was glued to the top of a 10×2×0.5 mm^3 STO single crystal, which is thermally anchored to a cryostat. A differential thermocouple was also glued to the surface of the crystal for accurate determination of the thermal gradient, see Figure S6 for a scheme of the assembly.
Driving a current through the ceramic resistor stabilizes a temperature difference between the thermocouples placed on the surface of the sample. Repeating this procedure several times, it is possible to find a linear relationship between the power dissipated by the resistor through the sample and the temperature difference. The experiments were done under vacuum ($P<10^{-4}$ mbar).
We took special care not to generate thermal gradients that were too high, that could break the linearity between the heat flux through the sample and the thermal gradient itself. Considering the dimensions of our samples, we kept the temperature difference between the thermocouples less than 1.5 K (Figure S6 b).

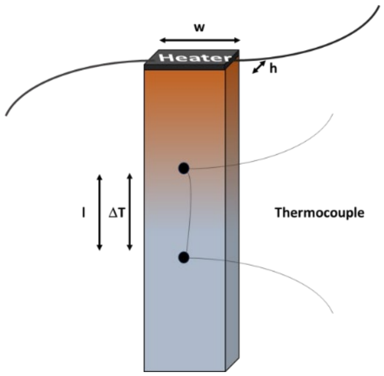

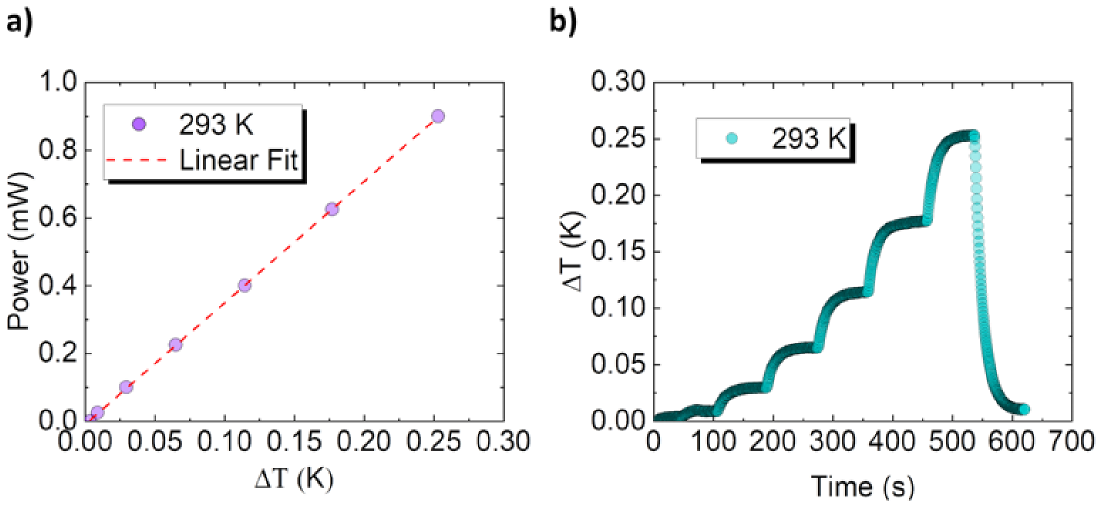

Figure S6. Top: Schematic representation of the assembly used to measure the thermal conductivity of STO and Nb:STO single crystals. Bottom: at 293 K a) the power dissipated vs the temperature difference recorded by the differential thermocouple, and b) temperature difference vs time recorded by the differential thermocouple attached to the surface of the crystal. Every step corresponds to a different current through the resistor.

The thermal conductivity can be obtained from the slope of the Power vs ΔT (Figure S6a) plot using the dimensions of the crystals:

$$\frac{dP}{\mathrm{d}\,\Delta T} \cdot \left(\frac{l}{w\,h}\right) = \kappa$$

The very good linearity in the $\Delta T<1$ K regime, ensures an accurate determination of the thermal conductivity of the samples. The results of the thermal conductivity at different temperatures are shown in Figure S7.

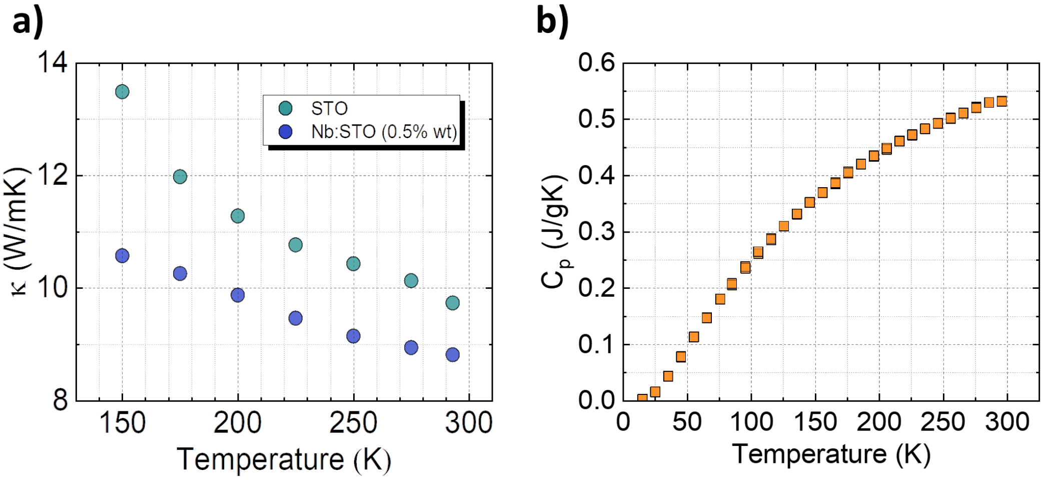

Figure S7. a) κ vs T plot for STO and Nb:STO (0.5 % wt) single crystal substrates. b) Cp vs T for STO.

Heat capacity measurements were performed in single crystal STO (Fig. S7b) in a PPMS of Quantum Design. The results of κ and C_p were used as fixed parameters in the FDTR fittings.

Frequency domain thermoreflectance.

The cross-plane thermal conductivity of the devices was measured by Frequency Domain Thermoreflectance (FDTR). For these measurements, a laser (λ=405 nm, modulating f=1kHz–50 MHz, Gaussian variable spot sizes 1/e2 radius ≈ 3.7 and 10.5 mm) is focused on the surface of the 60-nm thick layer of Au of the metal pads, which produces an oscillatory modulation of the surface temperature. This temperature change is probed by a laser beam (λ=532 nm) through a change in surface reflectivity. In our setup, the probe beam is split before reaching the sample to work as a reference signal, improving the signal-to-noise ratio at low frequencies and compensating phase-shift offsets from beam paths and electronics (see Figure S8). A similar setup is described in detail in reference Ref. [36].

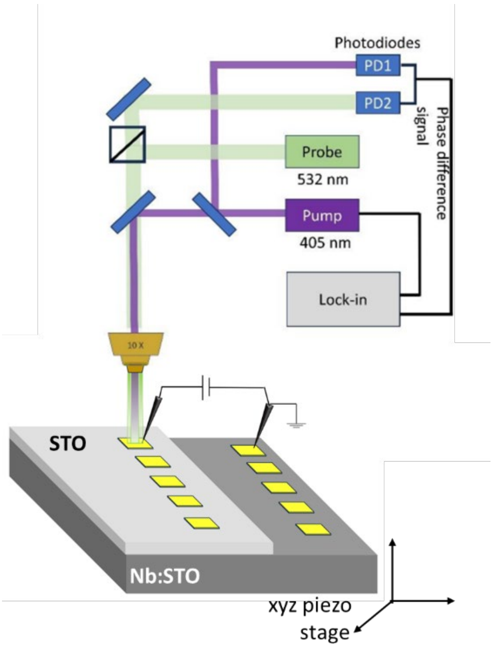

Figure S8. Simplified scheme of the FDTR setup used in this work to measure the metal/oxide thermal boundary resistance of the RS devices.

The thermal properties of the sample are obtained by fitting the phase data to an analytical solution of the heat diffusion equation, in a multilayer model as described in Ref.[35]. For minimizing the number of fitting parameters in the model, we determined most of them from independent experiments. For instance, the electrical resistivity of the Au/(Pt,Cr) top electrodes was measured in co-deposited samples, and used the Wiedemann-Franz law to obtain its thermal conductivity. The heat capacity, C_p, of the Au transducer was obtained from the literature[Ref. 37].The thermal conductivity and heat capacity of the substrates were measured in independent experiments, as described in the previous section. For the STO film, we assumed the same C_p as the substrate. Finally, epitaxial growth of $SrTiO_3$ on such a structurally and chemically similar substrate as Nb:$SrTiO_3$ results in a very low interfacial TBR (≈1-2 $m^2K\ GW^{-1}$); sensitivity experiments (Figure S9) confirm the almost negligible effect of a small variation of this parameter on the fitting.

We performed sensitivity analysis for estimating the uncertainty of our FDTR measurements. We define the sensitivity of the phase signal to a fitting parameter *x* as (the phase in radians):

$$S_x = \frac{d\phi}{d\ln x}$$

To decouple the sensitivity for the parameters of interest ($k_\perp$, thermal boundary resistances, etc.) we combined experiments with different spot sizes, between 1 MHz to 50 MHz.

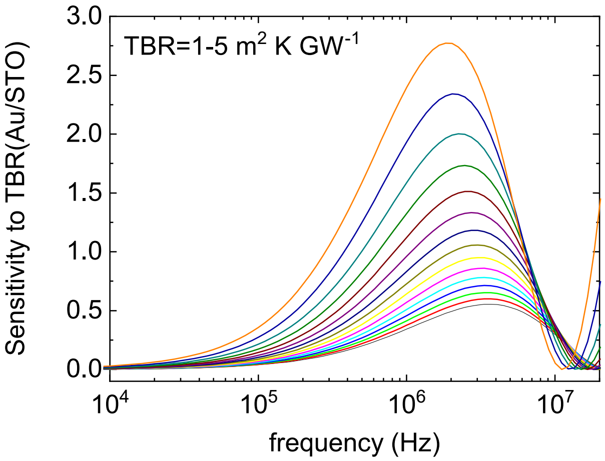

Figure S9. Phase sensitivity analysis of the TBR between a (60/5) nm thick film transducer of Au/Pt and a 35 nm thick $SrTiO_3$ film on a 500 μm thick Nb:STO substrate. The parameters of the film and transducer are listed on Table S1. The maximum sensitivity of the FDTR signal to metal/oxide TBR occurs from 0.1 MHz to 10 MHz..

The parameters used in the fittings are shown in Table S1.

TABLE S1: Typical values of the parameters determined from independent experiments and used for the phase/frequency fitting of the FDTR experiments. The value of TBR refers to the interface between the film and the substrate, which is very low due to the structural and chemical similarity, and it is kept constant during the fittings of the metal/oxide TBR.

	C_p (MJ $K^{-1}m^{-3}$)	k (W $m^{-1}K^{-1}$)	thickness (nm)	TBR (m^2 K GW^{-1})
**Au/Pt transducer**	2.5	61.44	65	
**Au/Cr transducer**	2.5	60.60	65	
**SrTiO3 film**	2.72	2	36.65	1-2 (STO/Nb:STO)
**Nb:STO substrate**	2.72	8.81	5×10^5	

In this way, the only fitting parameters are the TBR of the metal/oxide interface, and the κ of the STO film itself. Both magnitudes are correlated, and it is difficult to extract an accurate absolute value for each of them from the phase curve fitting, especially when working with nanometer-thick films, as in this case. Thus, we first measured κ of the substrate by the steady state method (Fig. S7a), and then performed FDTR experiments to obtain the metal/STO TBR. Using this TBR, we then measured the thermal conductivity of the 35 nm thick films of STO used in our devices. A value of $\kappa\approx2$ W $m^{-1}K^{-1}$ was estimated in this way, similar to previous reports for STO films with similar thickness [Ref. 38 in manuscript], and kept constant during the fittings. Following this procedure, the metal/oxide TBR is the only fitting parameter of the $\phi(\omega)$ curves of the FDTR experiments.
